# Measuring What Works: An Impact Evaluation of Women’s Groups on Maternal Health Uptake in Rural Nepal

**DOI:** 10.1371/journal.pone.0155144

**Published:** 2016-05-23

**Authors:** Sheetal Sharma, Edwin van Teijlingen, José M. Belizán, Vanora Hundley, Padam Simkhada, Elisa Sicuri

**Affiliations:** 1 Faculty of Health and Social Sciences, Bournemouth University, Dorset, United Kingdom; 2 Institute for Clinical Effectiveness and Health Policy, Buenos Aires, Argentina; 3 Centre for Public Health, Liverpool John Moores University, Liverpool, United Kingdom; 4 ISGlobal, Barcelona Ctr. Int. Health Res. (CRESIB), Hospital Clínic—Universitat de Barcelona, Barcelona, Spain; 5 Health Economics Group, Department of Infectious Disease Epidemiology, School of Public Health, Imperial College London, London, United Kingdom; Hunter College, UNITED STATES

## Abstract

**Background:**

There is a need for studies evaluating maternal health interventions in low-income countries. This paper evaluates one such intervention designed to promote maternal health among rural women in Nepal.

**Methods and Results:**

This was a five-year controlled, non-randomised, repeated cross-sectional study (2007, 2010, 2012) of a participatory community-based maternal health promotion intervention focusing on women’s groups to improve maternal health services uptake. In total 1,236 women of childbearing age, who had their last child ≤ two years ago, were interviewed. Difference-in-Difference estimation assessed the effects of the intervention on selected outcome variables while controlling for a constructed wealth index and women’s characteristics. In the first three years (from 2007 to the 2010), the intervention increased women’s likelihood of attending for antenatal care at least once during pregnancy by seven times [OR = 7.0, 95%CI (2.3; 21.4)], of taking iron and folic acid by three times [OR = 3.0, 95%CI (1.2; 7.8)], and of seeking four or more antenatal care visits of two times, although not significantly [OR = 2.2, 95%CI (1.0; 4.7)]. Over five years, women were more likely to seek antenatal care at least once [OR = 3.0, 95%CI (1.5; 5.2)], to take iron/folic acid [OR = 1.9, [95% CI (1.1; 3.2)], and to attend postnatal care [OR = 1.5, [95% CI (1.1; 2.2)]. No improvement was found on attending antenatal care in the first trimester, birthing at an institution or with a skilled birth attendant.

**Conclusion:**

Community-based health promotion has a much stronger effect on the uptake of antenatal care and less on delivery care. Other factors not easily resolved through health promotion interventions may influence these outcomes, such as costs or geographical constraints. The evaluation has implications for policy and practice in public health, especially maternal health promotion.

## Introduction

Health and development interventions in low-income countries (LICs) need high-quality evaluation [1–3]. Although evidence-based practice is making inroads into public health, there are still too many programmes that lack impact evaluation [4–6].

The main question of impact evaluation is one of attribution: isolating the effect of the programme from other intervening factors and potential selection bias of participants [7–10]. Impact evaluation assesses the medium- to longer-term changes of selected outcomes that can be attributed to a particular intervention, such as a project, programme or policy. These may be both the intended and, unintended outcomes [9,11].

In 2007, Green Tara Nepal (GTN), a non-governmental organisation (NGO), designed and implemented a five-year intervention to improve maternal health uptake in rural Nepal. The intervention included participatory action approaches, health promotion and small incentives. In Nepal, there is inequitable access to maternal health services due to factors such as geography, caste, ethnicity, religion and corruption [12–15].

The GTN intervention was designed to fit national policies and incorporated maternal health promotion focusing on women’s groups [16–18]. Such health promotion empowers women to seek care when it is needed, which is particularly important in low-income settings [4,19].

This paper reports on the impact evaluation of GTN, selecting as surrogate maternal mortality outcomes factors such as antenatal care (ANC), skilled birth attendance (SBA) and institutional delivery. These outcomes were chosen because they are indirectly correlated with maternal mortality [20–22]. Difference-in-Difference (DiD), analysis was used to identify and measure the effect of the intervention on the selected outcomes [23–28].

## Methods

### Study area and population

The programme selected two districts (one intervention and one control) with similar socio-economic characteristics. The districts were close to Kathmandu, each with a total population of just under 9,000. Baseline population characteristics (wealth, age, and education) and health system characteristics were not statistically different between intervention and control districts [29–30]. For instance, the intervention area has a community hospital providing basic emergency obstetric care and two health posts. The control area has two health posts and a primary health care centre nearby (similar to the community hospital in intervention area).

The intervention villages were chosen from a few pre-selected districts not far from Kathmandu that were (a) safe to work at the time of Maoist rebellion (1996–2006), which was still on-going at the time the intervention was designed (2005/2006); (b) with the local maternal health needs identified by the community and (c) with political commitment towards change [31].

### Intervention design

Two health promoters, an auxiliary nurse midwife and a community medical assistant, established and supported women’s groups with enrolment running between 2006 and 2012. Groups comprised (a) women age 15 to 49 with at least one child under the age of two; (b) mothers-in-law; and (c) husbands, as the latter two are particularly influential in terms of women’s ability to access health services [19, 29, 32–33]. Family decision-making affects the first of the “3 delays” in the maternal morbidity and mortality conceptual framework, namely the delay in making the decision to seek care [34–35].

The intervention took advantage of existing regular monthly women’s group meetings, which were originally savings or literacy-based. The rationale was that women’s groups have the potential for scaling-up within existing social systems, where community and health institutions are working in synergy to improve access to quality maternal and newborn health services [36]. Once the GTN intervention was implemented, its groups met regularly (monthly) for health promotion activities. The evidence-based maternal health promotion was designed by GTN and maternal health researchers and delivered by GTN health promoters. Participatory activities with visual cards and role-playing were conducted on for example ANC, iron/folic supplementation, danger signs of pregnancy, safe delivery and postnatal care (PNC). The implementation consisted of 24 sessions of health promotion, each one lasting one hour. In 2010, there were 37 groups (of 12 daughters-in-law, two men and 23 mixed mothers-in-law/daughters-in-law) reaching over 1,100 people.

By 2012, there were 46 groups (11 daughter-in-law groups, two male only groups, and 33 mother-in-law and daughter-in-law groups) reaching over 733 people.

GTN’s prerogative was to include all castes and empower lower castes to attend. The groups were generally mixed-caste, however, in certain areas, (where not all castes are represented) groups consisted of only one or two castes (S2 Fig).

The GTN health promoters supported the existing health system of sub-health posts by health communication training with mother-child health workers, traditional healers and hospital staff (e.g. neonatal training); and mobile clinics visits to outlying areas.

The intervention included small individual incentives consisting of a baby blanket awarded on completion of four ANC visits, safe delivery kits available at subsidised prices and other goods of the value of less than ten US cents. These incentives aimed to encourage group attendance and health- care seeking behaviour.

Finally, the intervention was flexible; at each stage, barriers were identified; solutions incorporated and the intervention was reapplied to meet the local needs [31].

### Evaluation design

This was a five-year controlled non-randomised intervention. Surveys for its evaluation took place at three points in time (repeated cross-sections): before the intervention started in 2007 (baseline), after 2½ years, in 2010, and after five years in 2012. Trained fieldworkers conducted survey interviews. Surveys were conducted in four villages, two in the intervention and two in control districts. The two villages of the intervention district were the ones where the intervention took place; the two villages of the control area were very similar to the intervention villages in terms of health system characteristics and socio-economic conditions. Within the district, the intervention was carried out in two villages selected based on (a) the presence of an adequate health system infrastructure, specifically a community hospital basic emergency obstetric care and two health posts; (b) having obtained approval from the village head; (c) the willingness to collaborate of the local health staff; (d) being neither the richest nor poorest in the district. The list of all women in the four villages (N = 474, 484 and 463, in the first, second and third survey, respectively) meeting inclusion criteria (having at least one child under the age of two years at the moment of survey) was compiled for both the intervention and the control areas. Hence all women meeting the inclusion criteria in the area determined the sample size, and the sampling frame covered the total sample. Women were then approached and interviewed at home. If women were not available on the first home visit, interviewers returned on several occasions, and after the third negative attempt women were dropped from the list. Health and socio-economic data were collected on individual, household and village level predicators/indicators using a structured questionnaire.

All women who met the inclusion criteria, not only those who participated in the groups, were eligible for this evaluation: the evaluation aimed to capture positive ‘spillover’ effects as any community-based programme can have an impact not only on the women directly receiving the intervention but also on the overall community [37,38].

The Nepal Health Research Council (NHRC) granted ethical approval for the study. The study aims were verbally explained to participants, as well as the consequences of their involvement and the right to withdraw at any point. After verbal consent all data were recorded anonymously.

### Data analysis

Descriptive analysis included demographic, cultural and socio-economic characteristics as well as maternal outcomes data and decision-making. DiD assessed the individual probability of engaging in each of the outcome variables and measured the difference in each outcome between intervention and control groups and before and after treatment while controlling for potential explanatory variables.

Logistic multivariate regressions were applied. Control variables, in addition to the ones representing the impact of the intervention, were chosen based on the literature and from previously published Demographic Health Studies data based studies [12,39].

With the aim of evaluating the intervention at two points in time, two different types of regressions were estimated: (a) regressions on the sample of women interviewed at baseline (year 2007) and at midline (year 2010); (b) regressions on the sample of all women in the study, including baseline, midline and final evaluation (years 2007, 2010 and 2012). The former (a) permitted the research team to assess the effects of the intervention after 2 years and the latter (b) to evaluate the overall impact of the intervention after 5 years from start.

Specifically, the regressions from baseline to midline were:

Yni=β0+β1interventioni+β2afteri+β3after*interventioni+β4age+β5wealthindexi+β6educationi+β7parityi Eq [1]

The regressions from baseline to final evaluation were:

Yni=β8+β9interventioni+β10afterafteri+β11afterafter*interventioni+β12age+β13wealthindexi+β14educationi+β15parityi Eq [2]

Where *i = 1*,*…*,*N* is indicator of each women participating in surveys. *Y*_*n*_ are the binary response variables and *n* indicates:

- *(n = 1)* ANC attendance at least once during whole pregnancy;
- *(n = 2)* ANC attendance at least once during first trimester;
- *(n = 3)* at least four ANC visits. The WHO recommends a minimum of four ANC visits and that the first one should be within the first trimester of pregnancy [40];
- *(n = 4)* presence of a SBA during delivery; SBA in Nepal was defined as nurse-midwives, auxiliary nurse-midwives and obstetricians. The following groups were, excluded: traditional birth attendants, health attendants, medical students as they are not classified by WHO as SBA [41];
- *(n = 5)* institutional delivery (ID, including at hospital, primary health centre, private hospital or clinic. This was chosen as an outcome because it is a recognised strategy to improve maternal child health [42–44];
- *(n = 6)* attending PNC defined as mother and newborn being seen 24 hours after birth, as 60% of maternal deaths in the low and middle-income countries occur postpartum [45–47]; and
- *(n = 7)* taking iron and folic acid; in Nepal, supplementation is provided at government health facilities [39] to prevent anaemia and neural tube defects [48].
  The control variables were:
  - Intervention (treat) {Eqs [1] and [2]} denotes the observations of the two groups: intervention and control;
  - After {Eq [1]} denotes time, before (baseline) and after intervention started;
  - After*intervention (treat-after) {Eq [1]} identifies the group of observations belonging to the intervention group after the intervention started as compared to the remaining observations (namely all observations belonging to control group and intervention group before the programme started), its estimated coefficient, $\hat{\beta }$_*3*_ represents the impact of the intervention [49].
  - Afterafter {Eq [2]} identifies the observations collected both in the midline and in the final evaluation as compared to baseline. As for the variable after in Eq [1], this variable also represent time;
  - Afterafter*intervention (treat-afterafter) {Eq [2]} identifies observations belonging to the intervention group at the midline and at the final evaluation as opposed to baseline and to all the observations in the control group at any time. Its estimated coefficient, $\hat{\beta }$_*11*_ represents the impact of the overall intervention [49].
  - Age = {Eqs [1] and [2]} represents the age of the individual.
  - Wealth index {Eqs [1] and [2]} is extracted from a series of assets owned (see details below). This variable tests the hypothesis of maternal attendance/compliance depending on women’s socio-economic status [50–52]. This variable was included instead of caste due to the statistically significant relationship between the two variables both in the midline and in the overall evaluation (Respectively: Pearson chi^2^(14) = 326.14; Pr = 0.00 and Pearson chi^2^(14) = 424.75 Pr = 0.00).
  - Education = {Eqs [1] and [2]} indicates women’s level of education.
  - Parity = {Eqs [1] and [2]}.

S1 Table further describes control variables.

### Wealth index construction

The wealth index was constructed using Principal Component Analysis (PCA) among a number of assets owned by women’s families [53–54]. PCA is commonly used when household expenditure or income data are not available [55]. Assets included in the PCA were: owning a bicycle, motorcycle, goat or car, type of access to hygienic facilities (source of drinking water, type of toilet), number of rooms in house, and materials used in the dwelling (materials used for flooring, walls, roofing). A description of variables included in the PCA is provided in S1 Table. In S1 Fig we present the distribution of wealth according to high, middle and low castes, according to the published definition of caste [39,56]. High caste (= 1) included: Brahmin, Chhetri, Newar; middle caste (= 2) included: Tamang middle, or low (= 3): Newar Dalit, Balami, Dalit and others (Christian or Muslim), S1. In S1 Fig the zero represents the median of the wealth index ranking. The distribution is skewed to the left due to the poverty level of this population.

Data were analysed with STATA™ version 11.0 (Stata/SE 11.0 Stata corporation, College Station, TX, USA).

## Results

A total of 1,236 women (611 in the control and 625 in intervention area) completed the surveys, with an overall average response rate of 87%. Specifically, N = 412 women participated in the first, N = 421 in the second and N = 403 in the third survey (S2 Fig). Overall, the mean age of respondents was 25.4 ± 5.1 years, the mean age of marriage 19.6 ± 3.3 years and the mean age at their first pregnancy 20.9 ± 3.2 years. The main occupation of respondents was either housewife or farmer (89.5%). Most women were multigravida (56.1%) and 43.9% were primigravida (Table 1).

**Table 1:** Characteristics of respondents (intervention/control).

		Control			Intervention			
Characteristic used as denominator	Baseline2007	Midline 2010	Final 2012	p-value*	Baseline2007	Midline2010	Final 2012	p-value*
N	204	204	203		208	217	200	
**Religion**		%		0.0014		%		0.0042
Buddhist	34.8	18.1	31.0		31.7	22.6	18.5	
Hindu	62.3	80.4	68.0		66.4	74.7	77.5	
Other (Christian, Muslim)	2.9	1.5	1.0		2.0	2.8	3.0	
**Caste/Ethnicity**				0.2048				0.0358
Brahmin	10.3	10.8	6.9		19.2	13.8	13.5	
Chhetri	20.6	18.1	17.2		14.9	7.8	12.5	
Tamang	38.2	35.8	38.4		39.9	51.2	40.5	
Newar non Dalit	25.5	27.5	26.1		14.4	13.4	19.0	
Newar Dalit	1.5	2.5	2.5		1.4	0.5	1.5	
Dalit	1.5	2.0	2.5		1.9	3.2	3.0	
Balami	0	0	0		6.3	5.5	7.0	
Other (Gurung etc.)	2.5	3.4	6.4		1.9	4.6	3.0	
**Age of marriage (yrs**^**1**^**)**				0.0173				0.0165
15–19	50.0	52.9	41.4		60.6	60.4	48.5	
20–24	38.4	40.7	45.3		37.0	35.0	43.5	
25–29	10.8	5.4	10.3		1.9	4.2	7.5	
30 and above	2.0	2.0	3.0		0.5	0.5	0.5	
**Age of 1**^**st**^ **pregnancy**				0.0135				0.0001
14–19	37.7	37.3	27.6		51.4	37.8	31.5	
20–24	47.1	52.0	52.7		42.8	52.5	53.5	
25–29	13.7	8.8	15.3		4.8	8.7	13.0	
30 and above	1.5	2.0	4.43		2.0	2.0	2.0	
**Literacy**	64.2	77.0	70.4	0.0187	66.4	73.3	81.0	0.0037
**Education**				0.1269				0.0025
None	43.6	31.4	33.0		39.9	33.2	24.5	
Primary	28.4	41.7	34.5		31.7	42.9	38.0	
Secondary and higher	27.9	27.0	32.5		28.4	24.0	37.5	

^1^ Yrs–Years

*p-values are based on Kruskal Wallis test to compare each categorical variable across time.

In the five-year period, the characteristics of the sample changed, the number of Buddhists decreased, while Hindus remained the majority. At baseline, most women were married before the age of 20; this proportion fell to 40.4% in the control group and to 48.5% in the intervention group in last survey. Literacy rates increased steadily (Table 1).

In terms of decision-making, over time a larger proportion of women reported to have planned pregnancy and to have more autonomy over maternity care, for instance ANC and place of delivery self-decisions increased over the duration of the study (Table 2). These changes were statistically significant in the intervention area.

**Table 2:** Health care decision-maker in household: % change over time by area.

		Control				Intervention		
Outcome	Baseline 2007	Midline 2010	Final 2012	p-value	Baseline 2007	Midline 2010	Final 2012	p-value
N	204	204	203		208	217	200	
		%				%		
**Planned pregnancy**	69.1	81.3	82.2	0.5327	74.5	73.7	86.5	0.0001
**Decision-maker**								
**ANC**^**1**^	**164**	**174**	**170**	0.1378	**176**	**210**	**196**	0.002
Myself	39.0	41.4	54.1		39.2	58.5	50.0	
Husband	41.0	37.4	25.3		37.5	25.7	25.5	
Mother-in-law	13.4	6.3	3.5		15.3	6.6	2.6	
All Family Members/Jointly	6.1	14.9	17.0		8.0	9.1	21.9	
**Place of delivery**	**204**	**204**	**200**	0.4837	**208**	**217**	**199**	0.0001
Myself	42.2	42.2	52.0		42.3	58.1	39.7	
Husband	30.4	39.7	25.5		36.1	25.8	26.6	
Mother-In-Law/Grand Mother-in-law Father-in-law	24.0 2.94	8.8-	4.0-		16.41.4	9.70.9	4.0-	
All Family Members/Jointly	0.5	9.3	18.5		3.9	5.5	29.7	
**Health care in family**	**204**	**204**	**202**	0.0021	**208**	**217**	**200**	0.6204
Myself	38.2	24.5	36.1		37.0	41.9	45.0	
Husband	38.2	44.1	32.2		41.4	32.7	25.0	
Mother-in-Law/Grandmother-In-Law	17.2	6.4	6.4		19.7	14.3	4.0	
Father-in-law	5.9	15.2	3.0		2.0	5.1	1.5	
All Family Members/Jointly	0.5	9.8	22.3		2.0	5.0	24.5	

^1^ ANC—Antenatal care

At baseline 80.4% of women in the control area attended ANC compared to 84.6% in the intervention area. In the intervention area, from baseline to final evaluation, the proportion of women who sought ANC at least once significantly increased from 84.6% to 98.0%. The proportion seeking ANC in the first trimester significantly increased from 47.7% to 62.4%; those seeking ANC four or more times significantly increased from 67.3% to 81.0%. In addition, a greater proportion of women reported taking iron/folic acid (from 86.5% to 96.0%) and seeing a SBA (from 60.6% to 82.0%). Significant increases were also seen in seeking an institutional delivery (from 60.6% to 76.0%) and PNC (from 52.2% to 85.9%). Use of safe delivery kit significantly increases from 5.0% to 34.3%. Improvements were also registered in the control group but not all were significant (Table 3).

**Table 3:** Maternal health uptake (%).

	Control				Intervention			
Outcome	Baseline 2007	Midline 2010	Final 2012	p-value	Baseline 2007	Midline 2010	Final 2012	p-value
		%(N)				%(N)		
**Seeking ANC**^**1**^								
At least once	80.4 (160)	85.3 (174)	88.7 (178)	0.0653	84.6 (174)	96.8 (206)	98.0 (194)	0.0001
In the 1st Trimester	55.6 (204)	68.3 (204)	61.1 (203)	0.0558	47.7 (208)	61.2 (217)	62.4 (200)	0.0073
4 or more visits	59.3 (204)	64.2 (204)	70.0 (203)	0.0809	67.3 (208)	86.2 (217)	81.0 (200)	0.0001
**Seeking PNC**^**2**^	42.9 (204)	61.8 (204)	73.8 (203)	0.0001	52.2 (208)	76.9 (217)	85.9 (200)	0.0001
**Institutional birth**^**3**^	55.4 (204)	54.7 (203)	71.4 (203)	0.0005	60.6 (208)	66.7 (217)	76.0 (200)	0.0037
**SBA**^**4**^	55.9 (204)	63.2 (204)	75.4 (204)	0.0007	60.6 (208)	70.1 (216)	82.0 (200)	0.0001
**Iron/Folic Acid during pregnancy**	76.4 (203)	79.9 (204)	79.3 (203)	0.6457	86.6 (208)	94.5 (217)	96.0 (200)	0.0006
**Use of safe delivery kit**	11.5(87)	17.1 (76)	11.6 (43)	0.5327	5.0(80)	40.3(67)	34.3 (35)	0.0001

^1^ ANC—Antenatal care

^2^ PNC–Postnatal care

^3^ ID–Institutional delivery

^4^ SBA–Skilled birth attendant

### Impact of the intervention

Tables 4 and 5 show the estimated odds ratios (OR) for the mid and overall evaluations, respectively. The effect of the intervention is represented by the estimated values of the OR of the variables treat-after (midline evaluation) and treat-afterafter (final evaluation). From baseline to the midline there was an increase in women’s likelihood of attending ANC at least once during whole pregnancy by 7.0 times [OR = 7.0, 95%CI (2.3; 21.4)]. A significant increase was also seen in the probability of taking iron/folic acid [OR = 3.0, 95%CI (1.2; 7.9)]. The probability of seeking four or more ANC check-ups had doubled, but this increase was not statistically significant [OR = 2.2, 95%CI (1.0; 4.7)] (Table 4). Over the five years (from baseline to final term), women were three times more likely of seeking ANC at least once [OR = 3.0, 95%CI (1.5; 5.8)]. Women were nearly twice as likely [OR = 1.9, [95% CI (1.1; 3.2)] to take iron/folic acid, and once and a half times as likely to attend PNC [OR = 1.5, [95% CI (1.1; 2.2)].

**Table 4:** Difference in difference analysis of maternal health uptake (intervention and control) at the midline evaluation.

	Seeking ANC^1^ at least once	Seeking ANC in the 1st Trimester	Seeking ANC 4 or more times	Taking Iron/Folic Acid during pregnancy	SBA^2^	ID^3^	Seeking PNC^4^
**Observations**	832	714	832	831	832	830	832
**Treat**	1.3 (0.7; 2.4)	0.7 (0.4; 1.1)	1.2 (0.7; 2.0)	2.3 (1.2; 4.1)**	1.2 (0.7; 1.9)	1.2 (0.8; 2.0)	1.5 (0.9; 2.3)
**After**	1.2 (0.6; 2.2)	1.6 (1.0; 2.6)	0.9 (0.5; 1.5)	1.0 (0.6; 1.7)	1.3 (0.8; 2.0)	0.8 (0.5; 1.3)	2.3 (1.5; 3.6)**
**Treat-after**	7.0 (2.3; 21.4)**	1.2 (0.6; 2.4)	2.2 (1.0; 4.7)*	3.0 (1.2; 7.9)**	1.5 (0.7; 2.8)	1.7 (0.9; 3.3)	1.6 (0.9; 3.1)
**Wealth**							
Wealth 2	3.4 (1.9; 6.0)**	1.7 (1.1; 2.5)**	1.2 (0.8; 1.9)	2.6 (1.5; 4.4)**	2.7 (1.9; 3.9)**	2.2 (1.5; 3.2)**	1.7 (1.2; 2.5)**
Wealth 3	6.0 (2.3; 15.7)**	3.1 (1.9; 4.9)**	5.2 (2.6; 10.8)**	2.8 (1.3; 5.8)**	11.0 (6.3; 19.4)**	7.6 (4.6; 12.7)**	4.0 (2.5; 6.4)**
**Age**	0.9 (0.9; 1.0)**	1.0 (1.0; 1.1)	1.0 (1.0; 1.0)	0.9 (0.9; 1.0)**	1.0 (1.0; 1.0)**	1.0 (1.0; 1.0)	1.0 (1.0; 1.0)
**Education**							
Education 2	5.2 (2.7; 10.1)**	1.5 (1.0; 2.3)**	1.0 (0.6; 1.5)	4.0 (2.3; 7.0)**	2.0 (1.3; 2.9)**	2.0 (1.3; 2.9)**	2.1 (1.4; 3.0)**
Education 3	9.3 (3.1; 28.0)**	2.6 (1.6; 4.2)	2.0 (1.0; 3.9)**	10.1 (3.9; 25.8)**	4.7 (2.8; 8.1)**	4.3 (2.6; 7.1)**	4.5 (2.8; 7.3)**
**Parity**							
Parity 2	0.7 (0.4; 1.2)	0.6 (0.4; 0.9)**	0.6 (0.4; 0.9)**	0.5 (0.3; 1.0)**	0.5 (0.4; 0.8)**	0.53 (0.4; 0.8)**	0.7 (0.5; 1.0)**
Parity 3	0.5 (0.3; 0.9)**	0.6 (0.4; 0.9)**	0.6 (0.4; 1.1)	0.3 (0.2; 0.6)**	0.6 (0.4; 1.0)*	0.6 (0.4; 1.0)**	0.75 (0.5; 1.2)

^1^ ANC—Antenatal care

^2^ SBA–Skilled birth attendant

^3^ ID–Institutional delivery

^4^ PNC–Postnatal care

**pvalue<0.05

**Table 5:** Difference in difference analysis of maternal health uptake in the overall evaluation.

	Seeking ANC^1^ at least once	Seeking ANC in the 1st Trimester	Seeking ANC 4 or more times	Taking Iron/Folic Acid during pregnancy	SBA^2^	ID^3^	Seeking PNC^4^
**Observations**	1235	1086	1235	1233	1235	1233	1235
**Treat**	1.5 (0.8; 2.6)	0.7 (0.5; 1.0)	1.5 (0.9; 2.4)	2.4 (1.4; 4.2)**	1.3 (0.9; 2.0)	1.5 (1.0; 2.3)**	1.5 (1.0; 2.3)**
**Afterafter**	1.2 (0.9; 1.6)	1.0 (0.8; 1.3)	1.0 (0.8; 1.3)	0.9 (0.7; 1.2)	1.3 (1.1; 1.7)**	1.3 (1.0; 1.6)**	1.8 (1.4; 2.2)**
**Treat-afterafter**	3.0 (1.5; 5.8)**	1.2 (0.9; 1.7)	1.1 (0.7; 1.6)	1.9 (1.1; 3.2)**	1.0 (0.7; 1.5)	0.9 (0.7; 1.3)	1.5 (1.1; 2.2)**
**Wealth**							
Wealth 2	2.5 (1.6; 4.0)**	1.8 (1.3; 2.4)**	1.4 (1.0; 2.0)	2.6 (1.7; 4.0)**	2.6 (1.9; 3.5)**	2.2 (1.6; 3.0)**	1.7 (1.2; 2.3)**
Wealth 3	4.7 (2.2; 10.4)**	2.8 (1.9; 4.1)**	3.3 (2.0; 5.6)**	2.4 (1.4; 4.2)**	9.3 (5.9; 14.7)**	7.0 (4.6; 10.6)**	3.8 (2.6; 5.7)**
**Age**	0.9 (0.9; 1.0)**	1.0 (1.0; 1.0)	1.0 (1.0; 1.0)	1.0 (1.0; 1.0)**	1.0 (1.0; 1.0)	1.0 (1.0; 1.0)	1.0 (1.0; 1.0)
**Education**							
Education 2	4.7 (2.7; 8.1)**	1.4 (1.0; 1.9)	1.0 (0.7; 1.5)	3.3 (2.1; 5.1)**	1.9 (1.4; 2.6)**	1.9 (1.4; 3.0)**	2.2 (1.6; 3.1)**
Education 3	11.0 (4.2; 29.0)**	2.2 (1.5; 3.3)**	1.8 (1.1; 3.0)**	9.3 (4.6; 18.9)**	4.6 (2.9; 7.1)**	4.0 (2.6; 6.0)**	4.7 (3.1; 7.1)**
**Parity**							
Parity 2	0.6 (0.4; 1.0)*	0.7 (0.5; 1.0)**	0.7 (0.5; 1.04)	0.6 (0.4; 0.9)**	0.5 (0.3; 0.6)**	0.5 (0.4; 0.7)**	0.6 (0.4; 0.7)**
Parity 3	0.4 (0.3; 0.8)	0.6 (0.4; 1.0)**	0.7 (0.4; 1.09)	0.3 (0.2; 0.5)**	0.5 (0.3; 0.8)**	0.5 (0.4; 0.8)**	0.7 (0.4; 1.0)**

^1^ ANC—Antenatal care

^2^ SBA–Skilled birth attendant

^3^ ID–Institutional delivery

^4^ PNC–Postnatal care

**pvalue<0.05

No effect was seen in the midline, or in the overall evaluation, on attending ANC in the first trimester, seeking an institutional delivery and having a SBA (Tables 4 and 5). A high OR was found for four ANC visits (Table 4), but not in the overall evaluation (Table 5).

### Results on remaining covariates

Wealth was a significant factor explaining a high proportion of the variation in all the outcomes both in the midline and in the overall evaluation. In particular, being richer (3^rd^ tertile) compared to being poorer (1^st^ tertile) increased substantially the probability of having a SBA at birth by the midline [(OR = 11.0, 95% CI (6.3; 19.4)] and by the overall evaluation [OR = 9.3, 95% CI (5.9; 14.7)].

Relative to the construction of the wealth index, the first component extracted explained 20% of total variability in the population. The scores based on the first component were grouped into tertiles in S1 Fig, with the lowest (group 1) representing the poorest and the higher (group 3) representing the richest women. Age was a significant factor in determining whether women sought one antenatal visit and took iron/folic acid both at the midline and final evaluation. In both cases, being older lowered the probability of a positive outcome. Having higher education compared to no education increased the probability of all the outcomes considered. In particular, having secondary school or higher-level education increased the probability of attending ANC at least once in the midline [OR = 9.3, 95% CI (3.1; 28.0)] and overall evaluation [OR = 11.0, 95% CI (4.2; 29.0)].

In the intervention area (variable treat), women were more predisposed to seek an institutional delivery [OR = 1.5, 95% CI (1.0; 2.3)] and PNC [OR = 1.5, 95% CI (1.5; 2.3)] at any time. Women in the intervention area were 2.3 [OR = 2.3, 95% CI (1.2; 4.1) times more likely at midline to take iron/folic and 2.4 [OR = 2.4, 95% CI (1.4; 4.2)] by year 5. Over time (variable afterafter), women become increasingly more likely to have a SBA at birth [OR = 1.3, 95% CI (1.1; 1.7)], institutional delivery [OR = 1.3, 95% CI (1.0; 1.6)] and PNC [OR = 1.8, 95%CI (1.4; 2.2)], reflecting background changes. With increasing parity, the ORs for all outcomes remain significantly below 1, indicating a negative relationship between having more than one child and the attendance to any type of maternal health service (either ANC or PNC) or the adherence to any type of pregnancy intervention, such as folic acid and iron intake.

## Discussion

This evaluation showed that the health promotion intervention had a positive effect on the uptake of ANC (attending at least once), iron/folic acid intake and PNC, but not on institutional delivery. While there was a positive effect on ANC attendance at least once during pregnancy, no effect was seen on ANC attendance in the first trimester. This may be because women become aware of the pregnancy status later or there are cultural reasons for the pregnancy to be kept a ‘secret’ [33–34, 42, 57]. Often first-time mothers need to ask permission and money from her family to attend ANC [58].

ANC attendance reduced from the midline to the overall evaluation, suggesting that the intervention may have diminishing returns: it could be argued that the effect was stronger when moving from low coverage to a medium level beyond which marginal improvements started decreasing [59]. The same trend as for ANC at least once was seen for iron/folic acid supplementation [60].

The barriers to accessing institutional delivery may be due to distance and socio-cultural factors (e.g. not part of a family’s birth preparedness plans) that cannot be overcome by a community-based intervention. Other studies in Nepal have suggested socio-economic, financial and geographical obstacles to seeking delivery care [45,61]. Studies with a focus in similar interventions have found that women’s groups in LICs increase the knowledge of obstetric ‘danger’ signs but have little impact on SBA due to other barriers, such as the cost of reaching a facility [62–64].

Although ANC attendance and provision of iron/folic acid during pregnancy are targets likely to be achieved in a community-based intervention such interventions without additional resources (such as ambulances) may not be influential enough to increase SBA or institutional delivery. Whilst PNC uptake is generally low for similar reasons to ANC non-attendance [65–66]. According to descriptive statistics, coverage of PNC greatly increased over the five years, yet the intervention only had a positive and significant effect at the very end of the intervention.

However, during 2007–2010, improvements were not associated with the GTN intervention but were due to other determinants, as witnessed by the significance of the variable “time” in both midline and overall regressions. The progressive improvement in women’s level of education and empowerment within the household may have played a role. Empowerment increased for women in particular with regard to decision-making power for ANC and delivery care. However, empowerment was not included in the estimated models as the trend was captured both by time and education (education, age and parity were strongly correlated). Interestingly, the increased women’s empowerment could be attributed to the health promotion intervention itself. Complex relationships are likely to exist among education, empowerment, maternal outcomes and the health promotion intervention [67,68].

In the overall evaluation, the factor “time” (represented by variable “afterafter”) was significant for PNC, delivery care (SBA and institutional delivery) outcomes, highlighting that other factors, not the GTN intervention, played a role. For instance, women were more likely to attend care if they have high household wealth, higher levels of education and lower parity [57,69]. A wealth index was preferred to caste as a more refined measurement for its greater inclusivity as a socio-economic indicator.

These results suggest that health promotion groups improve access to maternal health when individual, socio-economic and environment (health system) conditions are addressed [70]. Moreover, for this rural LICs setting, a health promotion intervention facilitating behavioural change may be more suitable as opposed to a cash transfer scheme alone. Cash transfer schemes, such as the ‘Safe Mother Program' (*Aama*-Suraksha-Karyakram) maternity incentive scheme, are often not financially sustainable [71–72].

Measuring the effect of a community-based intervention is not straightforward because of confounding environmental factors. Therefore, a (quasi-) experimental design is needed to ascertain whether the changes or improvements are due to the intervention or to external factors [5,28,73]. Previous studies have evaluated community-based interventions through randomised controlled trials (RCTs), the gold standard methodology for measuring effectiveness [74]. However, RCTs are difficult to organise, expensive and often setting-inappropriate for up-scaling or replicating and difficult to set up and run in LICs [25,75].

There is a need for community-based interventions to be accompanied by proper evaluations. Such evaluations can take the guesswork out of policy-making by knowing what works, what does not work and why [76–79]. Given the quasi-experimental study design, DiD permitted an approximation as close to an assessment of the effect of the programme on the outcomes of interest. Previous studies have assessed the effectiveness of programmes whereby a DiD approach was applied to community-based interventions [24–25]. In terms of data analysis, the criteria for inclusion of variables in a multivariate model vary between problems and disciplines. We followed methodologists suggesting the inclusion of all relevant variables in the model regardless of their significance in order to control for as many as possible covariates [80]. This approach, however, led in some cases to wide standard errors in estimated parameters, but as control variables were based on the literature, the impact of irrelevant variables was limited.

For this evaluation, maternal health behaviour before and after the intervention was not independently measured but based on self-reporting, which may have led to issues of validity and to recall bias. However, it is unlikely that the quality of self-reporting was different between intervention and control groups.

A weakness of this study is that the data collected did not identify any social barriers (e.g. gender of health worker), financial or geographical barriers (e.g. travel distance) to healthcare uptake that might account for the changes found, or lack thereof, between the data collection points. Moreover, for any intervention to improve maternal care and leverage commitment that maternal health should be a human right, it is important to know whether it is sustainable, scalable and cost-effective [81].

## Conclusion

This impact evaluation suggested that the community-based health promotion intervention had a greater effect on the uptake of ANC than on delivery care. Other factors, not easily resolved through health promotion interventions, may influence the latter outcomes, such as costs or geographical constraints. However, all the selected maternal outcomes improved in time while, in parallel, women’s level of education and empowerment also increased, which may either have facilitated or be the consequence of the intervention. Interventions should prioritise impact evaluations to inform future health policies.

## Supporting Information

S1 Fig Wealth index distribution of participants. (DOCX)

S2 Fig GTN health promotion intervention in Nepal and its evaluation. (DOCX)

S1 Table Variables description and codification, overall evaluation. (DOCX)
