# Supplementary material for: Measuring What Works: An Impact Evaluation of Women’s Groups on Maternal Health Uptake in Rural Nepal
Source: PLoS One. 2016 May 23;11(5):e0155144. doi: 10.1371/journal.pone.0155144 (PMC4877042; doi:10.1371/journal.pone.0155144)
Supplement: S1 Fig — (DOCX) [file pone.0155144.s001.docx]

**S1 Fig**  Wealth index distribution of participants.
