# Supplementary material for: Measuring What Works: An Impact Evaluation of Women’s Groups on Maternal Health Uptake in Rural Nepal
Source: PLoS One. 2016 May 23;11(5):e0155144. doi: 10.1371/journal.pone.0155144 (PMC4877042; doi:10.1371/journal.pone.0155144)
Supplement: S2 Fig — (DOCX) [file pone.0155144.s002.docx]

**S2 Fig** GTN health promotion intervention in Nepal and its evaluation

## Evaluation of intervention

Survey performed in 2 villages chosen based on having similar characteristics as the villages of the intervention area

among all women having at least one child under the age of two years of age

Women included in the survey

Year 2007: (baseline) N= 204

Year 2010: N= 204

Year 2012: N= 203

Women included in the survey:

Year 2007: (baseline) N= 208

Year 2010 (midline): N= 217

Year 2012 (final): N= 200

Survey performed in the 2 intervention villages among

all women having at least one child under the age of two years

Groups formed between 2006 (N=37) and 2012 (N=46)

With over 1100 (2010) and 733 (2012) people participating in total

Intervention performed in 2 villages of the district chosen based on:

- Community hospital Basic Emergency Obstetric Care) and two health posts.
- Having approval from head of village
- Willingness to collaborate from the local health staff
- Neither the richest nor poorest villages of the district

1 intervention district chosen based on:

- Commutable distance (20km) from Kathmandu that were (a) safe to work at the time of Maoist rebellion (1996 – 2006); (b) with the local maternal health needs identified by the community and (c) with political commitment towards change

1 control district chosen based on:

- Commutable distance (20km) from Kathmandu and

- Similar characteristics to Intervention in terms of: Socio-economic characteristics (wealth, age and education) and health system characteristics.

## Intervention

## Control
