# Supplementary material for: Measuring What Works: An Impact Evaluation of Women’s Groups on Maternal Health Uptake in Rural Nepal
Source: PLoS One. 2016 May 23;11(5):e0155144. doi: 10.1371/journal.pone.0155144 (PMC4877042; doi:10.1371/journal.pone.0155144)
Supplement: S1 Table — (DOCX) [file pone.0155144.s003.docx]

**S1 Table 1** Variables description and codification, overall evaluation

| **Variable** | **Description** | **Values** |
| --- | --- | --- |
| Regressions | | |
| Intervention | Dichotomical variable | 0=control group; 1= intervention group |
| After (midterm regression) | Dichotomical variable | 0= time at baseline; 1= time at midterm |
| After*intervention (midterm regression) | Dichotomical variable | 0= control group at midterm; 1= intervention group at midterm |
| Afterafter (final regression) | Categorical variable | 0= time at baseline; 1= time at midterm; 2= time at final |
| Afterafter*intervention (final regression) | Dichotomical variables | 0=control group at final; 1= intervention groups at final; 2= intervention group at final |
| Age, representing the age of the individual at that point in time. | Continuous variable | 15 - 49 |
| Education, indicates women’s level of education | Categorical variable | 0= none; 1= primary; 2= Secondary and higher/tertiary |
| Parity, indicates number of children | Categorical variable | 1 = women are primipara; 2 = two children; 3 = three or more children. |
| Components for wealth index construction | | |
| Materials used for roofing | Dichotomical variable | 0= roof made of tin, hay, stone; 1= roof made of cement, tile, |
| Area of land owned | Dichotomical variable | 0= own land less than 3 Ropani (0.38 acres in the hills); 1= own land greater than 3 Ropani (0.38 acres in the hills) |
| Goat | Dichotomical variable | 0=none; 1= owns goats |
| Motorised vehicle | Dichotomical variable | 0=none; 1= owns a motorcycle |
| Car | Dichotomical variable | 0=none; 1= owns a car |
| Source of drinking water | Dichotomical variable | 0= non-piped source of water to the home (common or public piped water, well, borehole, rain water, surface water such as rain, dam, lake, pond, stream, stone tap or *dhara*); 1= piped source of water to the home |
| Type of toilet | Dichotomical variable | 0= pit latrine (with or without slab) or composting toilet; 1= owns a flush toilet (flush to piped sewer system, septic tank or pit latrine) |
| Number of rooms in dwelling | Ratio of room: person | Total household member/rooms in dwelling |
| Type of energy used to cook (natural) | Dichotomous variable | 0=none; 1= uses natural source directly (kerosene, wood, animal dung, coal, straw, shrubs, grass) |
| Type of energy used to cook (biogas) | Dichotomous variable | 0=none; 1= uses biogas (made from raw materials converted to gas: agricultural waste, manure, municipal waste, plant material, sewage, green waste or food waste) |
| Type of energy used to cook (LP gas) | Dichotomous variable | 0=none; 1= uses liquid petroleum gas (LP gas) |
| Type of energy used to cook (electricity) | Dichotomous variable | 0=none; 1= uses electricity to cook |
| Bicycle | Dichotomical variable | 0=none; 1= owns a bicycle |
| Mobile phone | Dichotomical variable | 0=none; 1= owns a mobile pone |
| Fridge | Dichotomical variable | 0=none; 1= owns a fridge |
| Computer | Dichotomical variable | 0=none; 1= owns a computer |
